# Post-traumatic growth in liver cirrhosis patients: a cross-sectional study on the roles of psychological resilience and fear of progression based on the stress-coping theory

**DOI:** 10.3389/fpsyg.2026.1636895

**Published:** 2026-03-10

**Authors:** Yanyan Wang, Fei Kong, Qing Zhang, Qianqian Wang, Lijuan Yang

**Affiliations:** 1 Bone and Joint Tumor Surgery Department, Shandong Provincial Hospital Affiliated to Shandong First Medical University, Jinan, China; 2 Gastroenterology Ward 1, Shandong Provincial Hospital Affiliated to Shandong First Medical University, Jinan, China; 3 Trauma Orthopedics Ward 1, Shandong Provincial Hospital Affiliated to Shandong First Medical University, Jinan, China; 4 Department of Obstetrics and Gynecology Ward 1, Shandong Provincial Hospital Affiliated to Shandong First Medical University, Jinan, China; 5 Nursing Department, Shandong Provincial Hospital Affiliated to Shandong First Medical University, Jinan, China

**Keywords:** fear of progression, influencing factors, liver cirrhosis, post-traumatic growth, psychological resilience

## Abstract

**Objective:**

To investigate the current status of post-traumatic growth in patients with liver cirrhosis, analyze the influencing factors of post-traumatic growth, and provide a theoretical basis for the development of targeted intervention measures.

**Methods:**

A total of 250 patients hospitalized with liver cirrhosis were selected using a convenience sampling method from the Departments of Gastroenterology and Infectious Diseases of three tertiary hospitals in Shandong Province, between May and November 2023. Data were collected using a general information questionnaire, Psychological Resilience Scale, Chinese-Posttraumatic Growth Inventory, and Fear of Progression Questionnaire Short Form. The influencing factors of post-traumatic growth were analyzed.

**Results:**

A total of 250 patients with liver cirrhosis were included in the analysis. The mean post-traumatic growth score was (60.92 ± 10.74), the mean psychological resilience score was (26.89 ± 5.59), and the mean fear of progression score was (25.84 ± 6.50). Pearson correlation analysis showed a significant positive correlation between post-traumatic growth and psychological resilience (r = 0.667, *p* < 0.05) and a significant negative correlation between post-traumatic growth and fear of disease progression (r = −0.178, *p* = 0.006). Multiple linear regression analysis showed that a higher educational level (t = 2.037, *p* = 0.043, 95%CI: 4.164 ~ 9.442), psychological resilience (t = 11.308, *p* = 0.000, 95%CI: 0.342 ~ 0.744), and fear of progression in the social and family dimension (t = −2.398, *p* = 0.017, 95%CI: −0.388 ~ −0.026) were the main influencing factors of post-traumatic growth.

**Conclusion:**

Post-traumatic growth in patients with liver cirrhosis is at a moderate level. Fear of disease progression, psychological resilience, and educational level are the main influencing factors of post-traumatic growth. Healthcare professionals should focus on patients with high levels of fear of progression, low psychological resilience, and lower educational levels, and implement targeted intervention measures early to improve post-traumatic growth and promote the physical and psychological well-being of liver cirrhosis patients.

## 1 Introduction

Post-Traumatic Growth (PTG) refers to the positive psychological changes and self-improvement that individuals experience after undergoing negative life events or traumatic situations. It involves active resistance and adaptation, manifested in positive reconstruction in dimensions such as Philosophy of life, interpersonal relationships, and personal abilities (Tu and Guo, 2010). Fear of Disease Progression (FoP) is the persistent worry and anxiety that patients experience during the treatment or remission phase of their illness. This concern centers on the recurrence, deterioration, and the resulting physical suffering, as well as social dysfunction. It is a crucial cognitive-emotional response in the disease coping process (Dankert et al., 2003). Psychological Resilience (PR) is the core psychological trait that enables an individual to maintain psychological stability, rapidly recover and adapt in the face of adversity, trauma, or significant stress, and to achieve personal growth amidst difficulties (Bui et al., 2023).

Liver cirrhosis is the end-stage of chronic liver disease caused by various etiologies, including viral hepatitis, alcoholic liver disease, and non-alcoholic fatty liver disease. Its pathological features include diffuse liver fibrosis, the formation of pseudolobules, and the proliferation of regenerative nodules (Ge, 2018). A meta-analysis published in 2025, which included 44 global studies, showed that the global incidence of liver cirrhosis in the general population is 1.3% (112 million cases), with rates of 2.8% in Africa, 1.6% in North America, 1.5% in Latin America, 1.1% in Asia, and 1.0% in Europe. In China, the incidence rate is 1.2%, with approximately 10 million new cases annually. Due to the large population in China, it is currently one of the countries with the highest burden of liver cirrhosis globally (Zamani et al., 2025). Recent studies predict that by 2027, 35–42% of chronic liver disease patients in China will progress to cirrhosis (Ying, 2020), and the 5-year survival rate of patients in the decompensated phase is only 30–40% (Ginès et al., 2021). As the disease progresses, liver cirrhosis patients may develop multi-organ failure, with the decompensated phase often accompanied by severe complications such as splenomegaly, ascites, hypoalbuminemia, jaundice, and gastrointestinal bleeding (Di, 2022). This not only significantly reduces the patient’s quality of life but also increases the medical burden. The progression of chronic liver disease to cirrhosis is characterized by a long disease course, recurrent episodes, and uncertain prognosis, all of which bring a heavy toll on the patient’s physical and mental health (Chen, 2008). Studies have shown that approximately 16.7% of liver cirrhosis patients experience moderate to severe depressive symptoms, and 20.3% have moderate to severe anxiety, both of which are positively correlated with disease progression, creating a “mental disorder-disease deterioration” vicious cycle (Hernaez et al., 2022). In contrast, PTG, as a positive psychological adaptation outcome, can help liver cirrhosis patients reconstruct their disease perception, optimize coping strategies, and improve treatment adherence and overall physical and mental health (Zeng et al., 2018). Existing studies have confirmed that PTG can enhance chronic disease patients’ disease management abilities and improve their quality of life through mechanisms such as strengthening psychological resilience, enhancing social support, and increasing self-efficacy (van Gils et al., 2022). However, the occurrence mechanism and influencing factors of PTG in liver cirrhosis patients remain unclear.

**Figure 1:**
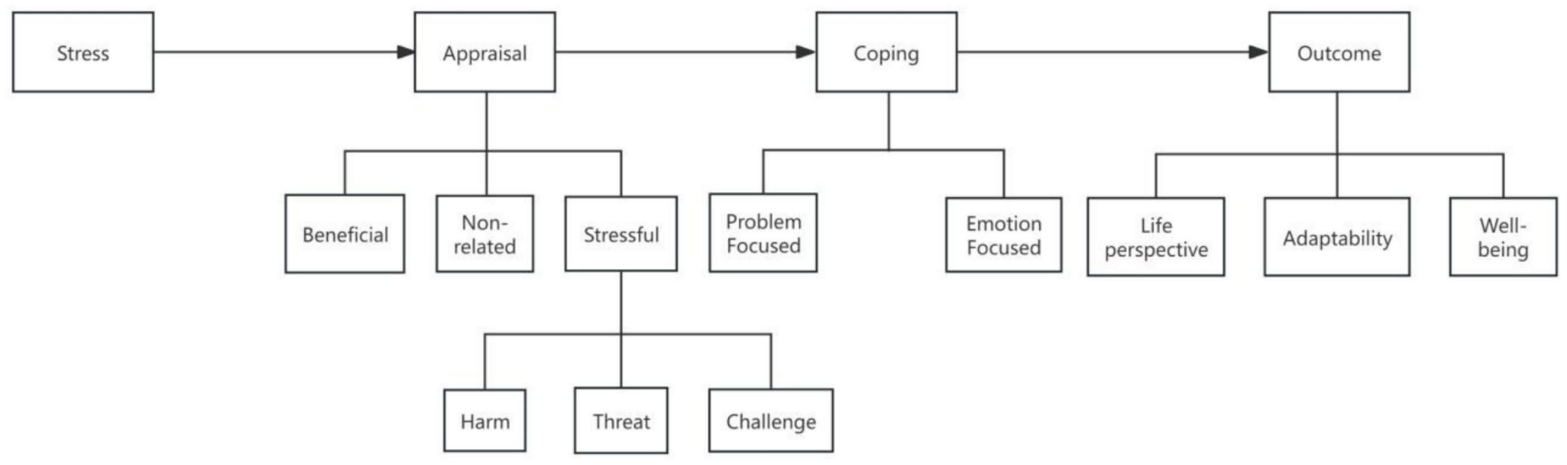
Stress and coping theory.

This study is based on the Stress and Coping Theory as the theoretical model. The theory was proposed by Lazarus and Folkman in 1984 (Figure 1) and includes three core variables: stressors, mediating variables, and stress responses (Lazarus and Folkman, 1984). Stressors refer to factors that threaten an individual’s survival or health. The individual’s performance and response to stressors depend on the mediating variables, such as cognitive appraisal and coping strategies. These mediating variables can be understood as determining the impact of the stressors on the individual (health/disease). Stress responses refer to the reactions individuals have to stressors, moderated by the mediating variables. Based on this theory, the following research hypotheses are proposed in this study:

*H1*: PR is positively correlated with PTG in liver cirrhosis patients, i.e., the higher the level of PR, the higher the PTG level in patients.

*H2*: FoP is negatively correlated with PTG in liver cirrhosis patients, and the social and family dimension of FoP has a more significant negative predictive effect on PTG.

In the appraisal-coping framework, there is a clear theoretical relationship between PTG, FoP, and PR. Liver cirrhosis, as the core stressor, first triggers the primary cognitive appraisal in patients. If patients assess their condition as a threatening event, they will experience FoP emotions (Zhang et al., 2025). In the secondary appraisal stage, PR, as a key internal resource, determines the patients’ choice of coping strategies: patients with high PR are more likely to adopt problem-focused coping strategies, transforming FoP into motivation for disease management. Through positive behaviors such as following treatment plans and seeking social support, they achieve PTG during the disease coping process (Li and Hu, 2022). On the other hand, patients with low PR are more likely to fall into emotion-focused coping, and excessive FoP can lead to sustained psychological stress, interfering with positive coping behaviors and thus hindering the development of PTG (Choi et al., 2023). In short, FoP is a direct emotional factor influencing PTG, while PR mediates or moderates the relationship between FoP and PTG by regulating cognitive appraisal and coping strategies.

Currently, most PTG-related research focuses on populations such as cancer patients, stroke patients, and those with post-traumatic stress disorder (Huang et al., 2025; Rao et al., 2025; Wen et al., 2025). Research on PTG in liver cirrhosis patients is relatively scarce, and the existing few studies have the following limitations: First, they have not clarified the intrinsic relationships between PTG, FoP, and PR based on a theoretical framework, making it difficult to reveal the mechanisms of PTG. Second, they neglect the disease specificity of liver cirrhosis—being a chronic progressive disease, its uncertainty, diverse complications, and long-term treatment pressures pose more complex psychological adaptation challenges for patients, making this group an important population for exploring positive psychological changes post-trauma. From a clinical perspective, liver cirrhosis patients have a high incidence of negative emotions, and PTG, as a potential protective factor, warrants exploration of its enhancement pathways, which could provide new ideas for clinical psychological interventions. From a theoretical perspective, studying liver cirrhosis patients could enrich the application of the stress-coping theory in the field of chronic liver disease and strengthen cross-disease research evidence on PTG.

Additionally, in China, the large number of liver cirrhosis patients and the heavy disease burden make it highly significant to explore the current status of PTG and its influencing factors, as this research can provide a theoretical basis for developing localized intervention measures. Based on this, the present study selects hospitalized liver cirrhosis patients as research subjects, investigates their PTG status, tests the aforementioned hypotheses, clarifies the correlation between FoP, PR, and PTG, and identifies the key factors of PTG. This study aims to provide theoretical evidence and clinical reference for constructing targeted psychological interventions, improving the physical and mental health of patients, and alleviating the disease burden.

## 2 Methods

### 2.1 Research design

This study adopts a cross-sectional research design and follows the reporting requirements of the Strengthening the Reporting of Observational Studies in Epidemiology (STROBE) guidelines (von Elm et al., 2008).

### 2.2 Sampling and recruitment process

A convenience sampling method was used to select liver cirrhosis patients hospitalized in the Departments of Gastroenterology and Infectious Diseases of three tertiary hospitals in Shandong Province from May to November 2023. The recruitment process was as follows: (1) The researchers communicated in advance with the head nurses and attending physicians of each department, explaining the study’s purpose, inclusion and exclusion criteria, and research process to gain clinical department support. (2) Healthcare professionals assisted in identifying hospitalized patients who met the clinical diagnosis of liver cirrhosis. The researchers then approached the patients, providing detailed information about the study background, objectives, participation duration, and potential risks and benefits. (3) For patients who expressed willingness to participate, eligibility was strictly verified according to the inclusion and exclusion criteria. After verification, the researchers communicated with the patients, obtained informed consent, and had them sign a written informed consent form. Subsequently, the patients were included in the study and questionnaires were distributed. The initial recruitment aimed for 270 participants, but the actual response rate was 265, with a response rate of 98.15%.

### 2.3 Inclusion and exclusion criteria

Inclusion criteria:

1. Age ≥18 years, regardless of gender;
2. Diagnosed with liver cirrhosis according to the clinical diagnostic criteria in the Guidelines for the Diagnosis and Treatment of Liver Cirrhosis (Chinese Medical Association, 2019), confirmed through imaging, laboratory tests, or pathological examination;
3. No severe mental disorders (such as schizophrenia, bipolar disorder, etc.) or cognitive impairments, and capable of understanding the questionnaire content;
4. Clear consciousness, Glasgow Coma Scale score >8, stable vital signs, and basic communication ability;
5. Voluntary participation in this study and signing a written informed consent form.

Exclusion criteria:

1. Explicitly refusing to participate or being uncooperative during the survey or withdrawing midway;
2. Unable to independently complete the questionnaire due to visual impairment, motor dysfunction, etc., and lacking qualified assistance (assistants are only allowed to read the questions, not provide hints);
3. Comorbidities with other severe organic diseases (such as late-stage malignancies, severe cardiovascular or cerebrovascular diseases), expected hospitalization duration <3 days, or unable to complete follow-up and questionnaire filling.

### 2.4 Sample size justification

The sample size for this study was calculated based on post-traumatic growth (PTG) as the dependent variable, using the sample size formula for multivariate analysis: n = μ^2^*α*/2*π*(1-π)/*δ*^2^, Where: PTG incidence rate (π) was taken as 0.33 based on reference (Xing and Zhang, 2023). The significance level (α) was set to 0.05, μα/^2^ = 1.96, the allowed error (δ) was set to 0.07. Using this, the minimum sample size required was calculated to be 173. Considering the potential for 20% invalid questionnaires, the adjusted minimum sample size was 217. This study included 250 participants, which meets the required sample size.

### 2.5 Ethical considerations

This study has been approved by the Ethics Committee of the School of Nursing and Rehabilitation, Shandong University, with the approval number 2023-R-062. The study was conducted in full compliance with the Declaration of Helsinki and relevant ethical guidelines. The process for obtaining informed consent is as follows: All participants were required to sign a written informed consent form. Before signing, the researchers provided a detailed explanation of the consent form to ensure that participants fully understood the voluntary nature of the study, the anonymity of participation, the scope of data usage, and privacy protection measures. Participants were clearly informed that they could withdraw from the study at any time without any conditions, and that withdrawal would not affect their regular medical services. All informed consent forms are securely stored by the research team for three years after the study’s completion. After three years, the forms will be destroyed promptly to ensure data security and privacy protection.

### 2.6 Research tools

#### 2.6.1 General information questionnaire for liver cirrhosis patients

The researchers developed the questionnaire based on relevant literature (Gu et al., 2025; Zhou et al., 2024) and clinical practice characteristics, which was reviewed and revised by three nursing experts (two chief physicians and one associate chief nurse). The survey covers two main dimensions: (1) Sociodemographic characteristics, including age, gender, marital status, residence (urban/rural), educational level, method of medical payment, occupational status, and average monthly income per family member. (2) Disease-related characteristics, including the duration of liver cirrhosis, number of hospitalizations, presence of complications and their types, and whether the patient has any comorbidities and the types of comorbidities.

#### 2.6.2 Fear of Progression Questionnaire Short Form (FoP-Q-SF)

In this study, the FoP-Q-SF translated and introduced into China by Wu et al. (2015), was used. The scale contains 12 items, covering two dimensions: physiological health and social-family. The physiological health dimension includes items 1, 2, 3, 5, 9, and 10, while the social-family dimension includes items 4, 6, 7, 8, 11, and 12. The scale uses a self-assessment method with scores ranging from 1 to 5, where 1 indicates “never” and 5 indicates “always.” The total score range is from 12 to 60. Based on the total score, three levels are defined: 12–25 points for low level, 26–46 points for moderate level, and 47–60 points for high level. A higher score indicates a stronger fear of disease progression. The Cronbach’s alpha coefficient of the scale is 0.85.

#### 2.6.3 Psychological resilience scale (CD-RISC-10)

The CD-RISC-10 used in this study is the revised version developed by Friborg et al. (2005). It consists of 10 items and one dimension. The scoring uses a Likert scale from 0 to 4, where 0 indicates “never” and 4 indicates “always.” The total score ranges from 0 to 40. A higher score indicates better psychological resilience. The Cronbach’s alpha coefficient for this scale is 0.905. In the Chinese population, the Cronbach’s alpha coefficient for the validated version of the scale is 0.984.

#### 2.6.4 Chinese-Posttraumatic Growth Inventory (C-PTGI)

In this study, the C-PTGI revised by Wang (2011), was used. The scale consists of 20 items, divided into 5 dimensions: (1) Philosophy of life (items 2, 5, 11, 13, 15, 19), with 6 items,(2) Personal strength (items 10, 12, 18), with 3 items, (3) New possibilities (items 9, 14, 16, 17), with 4 items, (4) Relationships with others (items 6, 8, 20), with 3 items, (5) Changes in self (items 1, 3, 4, 7), with 4 items. Patients use a 0 to 5 point rating scale for self-assessment, with a maximum score of 100. A higher total score indicates greater post-traumatic growth (PTG). The Cronbach’s alpha coefficient for the scale in this study was 0.915.

### 2.7 Data collection and data screening

#### 2.7.1 Data collection process

Before data collection, all researchers underwent standardized training, which included interpretation of the research protocol, guidance on filling out questionnaires, communication skills, informed consent procedures, and privacy protection requirements. Only after passing an assessment were the researchers allowed to participate in data collection. During the collection process, researchers went to the target departments, selected research subjects based on the inclusion and exclusion criteria, fully explained the study details to them, and obtained written informed consent before distributing the questionnaires. The questionnaires were completed and collected on-site, with the filling time controlled to be between 10 and 20 min. For patients who were unable to independently complete the questionnaires due to visual or motor impairments, the researchers read the items aloud, strictly adhering to the principles of “no suggestions, no leading.” The responses were recorded based on the patient’s verbal answers, and once completed, the researcher would review and verify the answers with the patient to ensure accuracy before collecting the questionnaires. A total of 265 questionnaires were distributed, and 255 were returned, yielding a response rate of 96.2%.

#### 2.7.2 Data screening process

After the questionnaires were returned, two researchers entered the data independently (using Excel to create a database). After the data entry was completed, a comparison was made to correct any errors, ensuring data accuracy. The next step involved screening for valid questionnaires. The criteria for invalid questionnaires were: (1) Item omission rate ≥10% (i.e., more than 2 items missing), (2) Identical answers for 5 or more consecutive items (e.g., all answered as “3”), indicating random answering, (3) Logical contradictions in answers (e.g., stating “no complications” but listing specific complications).

A total of 5 invalid questionnaires were excluded, leaving 250 valid questionnaires with an effective response rate of 94.3%.

### 2.8 Statistical methods

SPSS 26.0 was used to establish a database for data entry and analysis. For normally distributed continuous data, the mean ± standard deviation (SD) was used. Categorical and ordinal data were expressed as percentages (%) or frequencies. The impact of liver cirrhosis patients’ disease characteristics, sociodemographic data, fear of disease progression, and psychological resilience on post-traumatic growth was compared using independent samples t-test or one-way analysis of variance (ANOVA). Pearson correlation analysis was used to explore the correlation between disease fear progression, psychological resilience, and post-traumatic growth in patients. Multiple linear regression analysis was employed to identify the influencing factors of post-traumatic growth in liver cirrhosis patients. A *p*-value of <0.05 was considered statistically significant (two-tailed test).

## 3 Results

### 3.1 Basic characteristics of the study participants

Among the 250 liver cirrhosis patients who participated in this survey, the majority were aged 50–59 years, with 78 patients (31.2%) in this age range. The average age was (57.10 ± 11.26) years. Males accounted for 60% of the participants. In terms of marital status, the majority were married, with 221 patients (88.4%). The majority had an education level of junior high school or below, with 122 patients (48.8%). The most common medical payment method was urban resident insurance, with 129 patients (51.6%). Most participants were farmers, with 87 patients (34.8%). The majority of participants had a household monthly income per person between 5,001 and 10,000 RMB, with 147 patients (58.8%). The main caregivers were spouses, with 172 patients (68.8%). Significant differences in post-traumatic growth scores were found among liver cirrhosis patients based on marital status, residence, education level, medical payment method, occupation, household monthly income per person, disease duration, and whether they had comorbidities (*p* < 0.05). See Table 1 for details.

**Table 1:** Basic characteristics of the study participants.

Item	Group	Number (n)	Percentage (%)	PTG score (Mean ± SD)	t/F	*p*
Age (years)					1.289	0.269
	≤39	20	8	58.80 ± 11.8		
	40–49	44	17.6	60.45 ± 9.79		
	50–59	78	31.2	59.72 ± 10.83		
	60–69	69	27.6	61.30 ± 9.68		
	70–79	36	14.4	64.67 ± 12.04		
	≥80	3	1.2	60.01 ± 10.96		
Gender					1.090	0.277
	Male	150	60	61.52 ± 10.58		
	Female	100	40	59.72 ± 10.83		
Marital status					3.330	0.011
	Married	221	88.4	55.33 ± 11.59		
	Single	3	1.2	60.19 ± 10.40		
	Divorced	2	0.8	68.00 ± 16.97		
	Widowed	21	8.4	67.10 ± 11.26		
	Remarried	3	1.2	72.00 ± 12.77		
Place of residence					3.515	0.031
	Urban	78	31.2	63.14 ± 10.46		
	Town	87	34.8	61.06 ± 10.84		
	Rural	85	34	58.73 ± 10.57		
Education level					22.180	<0.001
	Junior high or below	122	48.8	56.68 ± 11.06		
	High school/technical	89	35.6	65.53 ± 8.67		
	College or higher	39	15.6	63.64 ± 8.78		
Method of medical payment					8.922	<0.001
	Self-paid	4	1.6	55.75 ± 3.40		
	Urban employee medical insurance	110	44	63.91 ± 10.21		
	Urban resident medical insurance	129	51.6	58.01 ± 10.57		
	Government-funded medical care	7	2.8	70.43 ± 5.38		
Occupation					2.431	0.024
	Farmer	87	34.8	59.0 ± 11.32		
	Worker	57	22.8	61.32 ± 9.47		
	Enterprise or institution employee	35	14	63.69 ± 9.11		
	Self-employed/freelancer	22	8.8	56.55 ± 11.88		
	Unemployed	6	2.4	58.83 ± 3.55		
	Retired	41	16.4	64.68 ± 11.29		
	Other	2	0.8	61.50 ± 9.19		
Family per capita monthly income					6.508	0.002
	<1,000	57	22.8	56.51 ± 11.73		
	1,000–5,000	147	58.8	62.15 ± 10.28		
	5,001–10,000	46	18.4	62.43 ± 9.59		
Primary caregiver					1.818	0.144
	Spouse	172	68.8	61.34 ± 10.97		
	Children	67	26.8	60.24 ± 10.00		
	Parents	4	2	65.80 ± 10.43		
	Other	5	2.4	52.33 ± 9.73		
Disease duration					3.022	0.030
	<1 year	85	34	58.81 ± 10.39		
	1–5 years	100	40	62.05 ± 11.11		
	6–10 years	32	12.8	64.59 ± 12.07		
	>10 years	33	13.2	59.33 ± 7.66		
Number of hospitalizations					1.470	0.232
	First time	82	32.8	61.91 ± 8.33		
	2–3 times	108	43.2	59.58 ± 11.39		
	>3 times	60	24	61.95 ± 12.26		
Comorbidity					0.423	0.672
	Yes	160	64	60.70 ± 11.11		
	No	90	36	61.30 ± 10.09		
Underlying diseases					2.755	0.006
	Yes	90	36	63.38 ± 10.24		
	No	160	64	59.53 ± 10.79		

### 3.2 Post-traumatic growth, psychological resilience, and fear of progression scores in liver cirrhosis patients

In this survey, the scores for post-traumatic growth, psychological resilience, and fear of progression in liver cirrhosis patients were normally distributed. The average PTG score was (60.92 ± 10.74). Among the dimensions of PTG, the average item score for the Relationships with others dimension was the highest, at (3.47 ± 0.63), followed by the Personal Strength dimension (3.33 ± 0.70). The Philosophy of life (2.81 ± 0.65) and New Possibilities (2.80 ± 0.65) dimensions had similar scores, while the Changes in self dimension had the lowest average item score, at (2.46 ± 0.72).

The psychological resilience score ranged from 11 to 39, with an average score of (26.89 ± 5.59). The fear of progression score ranged from 12 to 60, with an average score of (25.84 ± 6.50). See Table 2 for details.

**Table 2:** Levels of PTG, PR, and FoP in liver cirrhosis patients (*n* = 250, x̅ ± s).

Variable	Score range	Score range	Mean score	Average score per item	Rank
Total PTG score	0–100	22–84	60.92 ± 10.74	3.04 ± 0.57	—
Relationships with others	0–15	3–15	10.40 ± 1.89	3.47 ± 0.63	1
Personal strength	0–15	3–15	10.12 ± 2.11	3.33 ± 0.70	2
Philosophy of life	0–30	8–28	19.34 ± 3.28	2.81 ± 0.65	3
New possibilities	0–20	4–18	11.21 ± 2.58	2.80 ± 0.65	4
Changes in self	0–20	2–17	9.85 ± 2.87	2.46 ± 0.72	5
PR	0–40	11–39	26.89 ± 5.59	2.69 ± 0.56	
FoP	0–60	12–60	25.84 ± 6.50	2.15 ± 0.54	

### 3.3 Levels of PTG in liver cirrhosis patients

Among the 250 liver cirrhosis patients surveyed, the majority exhibited a moderate level of post-traumatic growth, with 202 patients (80.8%) falling into this category. See Table 3 for details.

**Table 3:** PTG grade in liver cirrhosis patients (*n* = 250).

Variable	Score range	*n*	Percentage (%)
Low level of PTG	0–35	3	1.2
Moderate level of PTG	36–70	202	80.8
High level of PTG	71–100	45	18

### 3.4 Pearson correlation analysis

Pearson correlation analysis showed that post-traumatic growth in liver cirrhosis patients was positively correlated with psychological resilience (r = 0.667, *p* < 0.05) and negatively correlated with fear of disease progression (r = −0.178, *p* < 0.005).

### 3.5 Multiple linear regression analysis results

PTG scores in liver cirrhosis patients were used as the dependent variable. Based on the results of univariate analysis and correlation analysis, which showed statistically significant differences or correlations with the dependent variable (such as marital status, residence, educational level, medical payment method, occupation, household monthly income per person, disease duration, comorbidities, FoP, and PR), multiple linear regression was performed. In the multiple linear regression model, educational level (with junior high school as the reference), PR, and the social-family dimension of FoP were included in the equation. These three variables accounted for 53.1% of the variance in PTG (adjusted R^2^ = 0.531). The equation for PTG in patients was: PTG = 18.157 + 2.635 * Educational Level + 1.161 * PR − 0.379 * FoP (Social-Family Dimension). The results are shown in Table 4.

**Table 4:** Multiple linear regression analysis results.

Variable	Regression coefficient	Standard error	Standardized regression coefficient	t-value	*p*	95%CI
Constant	18.157	5.787		3.138	0.002	39.965 ~ 55.482
High school education level	2.635	1.293	0.118	2.037	0.043	4.164 ~ 9.442
PR	1.161	0.103	0.604	11.308	0.000	0.342 ~ 0.744
FoP (social/family dimension)	−0.379	0.158	−0.122	−2.398	0.017	−0.388 ~ −0.026

### 3.6 Hypothesis testing

*H1*: It was hypothesized that PR is positively correlated with PTG in liver cirrhosis patients. The results showed a significant positive correlation between the total scores of PR and PTG (r = 0.667, *p* < 0.05), confirming that H1 is valid.

*H2*: It was hypothesized that FoP is negatively correlated with PTG in liver cirrhosis patients, and that the social-family dimension of FoP has a more significant negative predictive effect on PTG. Pearson correlation analysis showed a significant negative correlation between the total scores of PTG and FoP (r = -0.178, *p* < 0.005), confirming a negative association between the two, which supports the hypothesis.

### 3.7 Dimension prediction

In the multiple linear regression analysis, PTG total score was used as the dependent variable. After controlling for significant variables identified in univariate analysis and correlation analysis, the social-family dimension of FoP entered the equation (regression coefficient = −0.379, standardized regression coefficient = −0.122, t = −2.398, *p* = 0.017). The physiological health dimension did not enter the equation. This indicates that the social-family dimension of FoP has a significant negative predictive effect on PTG, which is more prominent. Therefore, H2 is valid.

## 4 Discussion

### 4.1 Current status of PTG in liver cirrhosis patients

In this study, the total PTG score for liver cirrhosis patients was (60.92 ± 10.74), slightly lower than the result found by Zhang et al. (2021) in a study on post-bladder cancer patients (61.47 ± 10.79). Considering the stress-coping theory and cultural context, the differences can be further analyzed from two perspectives: (1) The stress accumulation effect of marital status: In this study, the proportion of married patients was as high as 88.4%. In the Chinese “family-centered” cultural context, married patients not only bear the pressure of their own disease recovery but also take on responsibilities such as family caregiving and economic support. This dual-role pressure increases the cognitive appraisal burden, weakens the ability to mobilize positive coping resources, and thus suppresses the improvement of PTG levels. (2) Cognitive empowerment differences due to educational level: In this study, 48.8% of patients had a junior high school education or lower. Low educational levels not only limit patients’ ability to acquire and interpret disease information but also affect their efficiency in establishing positive cognitive frameworks. In contrast, patients with higher education are more likely to learn coping strategies through various channels. In comparison, lower-educated patients are constrained by cognitive resources and are more likely to fall into negative cognitive biases when faced with disease trauma, hindering the development of PTG.

### 4.2 Factors influencing PTG in liver cirrhosis patients

#### 4.2.1 Educational level

This study found a positive correlation between educational level and PTG, which is consistent with the conclusions of studies by Meng (2023) (on post-chemotherapy breast cancer patients of reproductive age) and Wen et al. (2025) (on post-surgery patients with primary liver cancer). From the perspective of the stress-coping theory, educational level does not directly affect PTG but exerts a mediating effect through cognitive appraisal and coping mechanisms. Patients with higher education possess stronger information processing abilities and are more capable of rationally assessing the impact of disease trauma, interpreting it as a manageable challenge rather than an uncontrollable disaster. This leads them to adopt problem-focused coping strategies, such as actively cooperating with treatment and learning disease management knowledge. Additionally, highly educated individuals are more likely to embrace psychological adjustment concepts, mobilize internal resources for cognitive restructuring, and ultimately promote PTG (O'Donovan and Burke, 2022). In contrast, patients with lower educational levels tend to experience excessive worry due to unclear disease cognition, often favoring emotion-focused coping strategies such as avoidance and denial. As a result, they struggle to achieve cognitive and psychological growth after trauma. This finding further supports the core role of cognitive appraisal in the stress-coping process and enriches the theoretical explanation of the relationship between educational level and PTG in chronic disease contexts. It is suggested that in clinical practice, differential interventions should be designed based on the educational level of patients, ensuring comprehensive assessment and tailored intervention strategies.

#### 4.2.2 Psychological resilience

This study confirmed a significant positive correlation between psychological resilience and PTG in liver cirrhosis patients, which is consistent with the findings of a related study on liver cancer patients by Yin (2024) and aligns with the core concept of “resilience resources” in the stress-coping theory. As a core psychological resource for individuals to cope with adversity, psychological resilience enhances positive coping abilities by regulating the cognitive appraisal process. Patients with high resilience are more likely to activate internal resources, such as an optimistic mindset and self-efficacy, when facing the chronic trauma of liver cirrhosis. This enables them to transform negative experiences associated with the disease into opportunities for self-growth (Lei et al., 2022). At the same time, psychological resilience can buffer the emotional impact of disease-related stress, reduce emotional exhaustion, and provide psychological energy for PTG. In the context of Chinese cultural characteristics, the mechanism of psychological resilience also implicitly reflects the cultural trait of “endurance and resilience.” Patients internalize this cultural concept, accumulate psychological capital in the fight against disease, and further promote post-traumatic growth (Guo et al., 2021). This finding expands the understanding of individual resources within the stress-coping theory and confirms the key empowering role of psychological resilience in the development of PTG in chronic liver disease patients. It is recommended that in clinical practice, for patients with low psychological resilience, timely targeted interventions should be implemented to enhance their resilience levels.

#### 4.2.3 Fear of disease progression

The results of this study showed a negative correlation between fear of disease progression and PTG, which is consistent with findings from related studies on cancer patients (Chen et al., 2016; Yao et al., 2020). Furthermore, the negative predictive effect of the social-family dimension of FoP was more significant. From the perspective of the stress-coping theory, FoP is essentially a result of negative cognitive appraisal. Patients with high FoP tend to interpret disease progression as a high-threat, low-controllability event, which leads to negative emotions such as anxiety and fear. These emotions occupy coping resources and inhibit positive cognitive restructuring. Considering cultural factors, in Chinese patients, FoP is more influenced by the social-family dimension. In the context of collectivist culture, patients are not only concerned about their own health but also fear the economic burden, caregiving pressure, and social stigma that the disease may impose on their families. This family-oriented fear strengthens the tendency for negative coping, further diminishing PTG levels (Stewart et al., 2024). However, the physiological health dimension of FoP did not show a significant predictive effect, which may be related to liver cirrhosis patients’ adaptation to physiological symptoms. Chronic illness leads patients to develop a certain tolerance to physical discomfort, and their psychological distress is more influenced by potential threats at the family and social level. This dimensional difference provides a reference for subsequent interventions.

#### 4.2.4 Others

In this study, although factors such as residence, medical payment methods, and income level were not included in the regression analysis, related studies have suggested that they may still be associated with PTG (Li et al., 2025; Lin, 2025). Residence and income level jointly determine the accessibility of medical resources and social support. Urban high-income patients are more likely to access quality medical services, psychological interventions, and social support, all of which can effectively reduce disease-related stress and facilitate PTG. In contrast, rural low-income patients, limited by a lack of resources, face greater disease-related stress that is difficult to buffer, thereby hindering PTG development. Medical payment methods influence economic stress, with patients having higher reimbursement rates from medical insurance being able to reduce the financial burden of illness, alleviating concerns about falling into poverty due to illness, and consequently being more likely to adopt positive coping strategies. It is important to note that these variables are not direct determinants of PTG. Instead, they may exert their influence through factors such as family and social support and inner peace. Therefore, PTG is influenced by multiple factors, not a single one. This suggests that when developing intervention strategies, we should consider multiple dimensions, including the patient’s physiological, psychological, cultural, and social resources.

## 5 Theoretical and practical contributions

### 5.1 Theoretical contributions

This study expands the application of the stress-coping theory in the field of chronic liver disease, confirming the core roles of cognitive appraisal, coping mechanisms, and psychological resilience in the development of PTG in liver cirrhosis patients. It provides empirical support for the stress-coping theory in the context of chronic diseases. Furthermore, it deepens the integrated analysis of the factors influencing PTG, incorporating variables such as educational level, FoP, and PR into a unified analytical framework. This study clarifies the correlation of these factors with PTG and offers new insights into refining PTG theory in the chronic disease context.

### 5.2 Practical contributions

This study suggests that clinical healthcare providers should consider educational level, PR, and FoP, with a particular focus on the social-family dimension, as key assessment indicators for PTG in liver cirrhosis patients. Special attention should be given to patients with low education, low resilience, and high FoP by establishing a focused care checklist. This individualized approach to psychological care will provide insights for improving PTG levels in these patients and will help enhance the rehabilitation of liver cirrhosis patients.

## 6 Strengths of this study

This study focuses on liver cirrhosis, a specific group within chronic liver diseases, and clearly identifies the influencing factors of PTG in this population. The research subjects are highly targeted, and the findings have direct reference value for psychological care in liver disease patients. Furthermore, this study integrates the stress-coping theory to analyze the Chinese liver disease population, providing empirical support for the localization of this theory in China. Additionally, the study refines the analysis of the dimensions of FoP, clarifying the special role of the social-family dimension. This provides more specific reference points for precision interventions. Compared to a general analysis of the relationship between FoP and PTG, the conclusions of this study offer more practical guidance.

## 7 Limitations and future research directions

### 7.1 Limitations

This study has certain limitations in its design. As a cross-sectional study, it can only reveal the correlation between variables and cannot determine whether improvements in psychological resilience directly lead to increased PTG levels. It is also difficult to clarify the dynamic interactions between variables. Moreover, the study did not perform mediation analysis, which may lead to incomplete representation of the interrelationships between variables. Additionally, the general information questionnaire was self-designed based on the literature, without using a formal scale development method for validation. This may pose a risk of overlooking some general factors. The study did not measure ASD/PTSD symptoms, which might have limited the understanding of the variables’ impact on the outcomes. Furthermore, the study did not include potential influencing factors such as the severity of liver cirrhosis, disease etiology, or spiritual/religious orientation, which could affect PTG levels through mediating or confounding effects, thus limiting the generalizability of the study’s conclusions. Finally, the sample was limited to a single center, with a small sample size concentrated in one region, which means the study results may not be generalizable to liver cirrhosis patients in different regions or under varying medical conditions.

### 7.2 Future research directions

Future studies should design longitudinal research and expand the follow-up period using tracking surveys to measure ASD/PTSD symptoms, dynamically observe the changes in PR, FoP, and PTG, and perform mediation analysis to clarify causal relationships and the sequence of effects between variables. This would further verify the long-term applicability of the stress-coping theory in chronic liver disease patients. Interventional studies can also be conducted based on the conclusions of this research to design integrated intervention programs. Randomized controlled trials could then be used to validate the effectiveness of these programs, providing a practical and standardized approach for clinical psychological care. Additionally, future studies should aim to expand the sample scope by conducting multi-center, large-sample studies that include patients from different regions, social-economic backgrounds, religious beliefs, and disease stages, to enhance the generalizability of the research findings. Qualitative interview methods could also be integrated to explore patients’ subjective experiences of PTG, thus complementing quantitative research and providing a more comprehensive basis for the development of intervention strategies.

## 8 Conclusion

This study found that educational level, psychological resilience, and FoP are the main influencing factors of PTG in liver cirrhosis patients. These factors operate through the cognitive appraisal and coping mechanisms in the stress-coping theory. High educational level and high PR promote positive cognition and coping, thereby enhancing PTG levels, while high FoP reinforces negative cognition and inhibits PTG development. Clinical healthcare providers should focus on patients with low education, low PR, and high FoP, implementing individualized interventions. By optimizing cognitive appraisal, strengthening psychological resources, and alleviating family-oriented fear, healthcare providers can promote PTG in patients and facilitate their disease recovery.

## Data Availability

The original contributions presented in the study are included in the article/supplementary material, further inquiries can be directed to the corresponding author.
